# Electromagnetic Field Exposure and Abortion in Pregnant Women: A Systematic Review and Meta-Analysis

**DOI:** 10.21315/mjms2023.30.5.6

**Published:** 2023-10-30

**Authors:** Morvarid Irani, Maryam Aradmehr, Mohammad Ghorbani, Roya Baghani

**Affiliations:** 1 Department of Midwifery, School of Nursing and Midwifery, Torbat Heydariyeh University of Medical Sciences, Torbat Heydariyeh, Iran; 2 Health Sciences Research Center, School of Health, Torbat Heydariyeh University of Medical Sciences, Torbat Heydariyeh, Iran; 3 Department of Midwifery, School of Nursing and Midwifery, Shahroud University of Medical Sciences, Shahroud, Iran

**Keywords:** electromagnetic field, pregnancy, abortion, miscarriage

## Abstract

This study examined the effects of exposure to electromagnetic fields (EMF) on pregnancy and the risk of miscarriage. We performed a systematic search for relevant studies published to August 2021 in the medical databases of PubMed, CINAHL, Scopus, Web of Science, Google Scholar and Cochrane Library. The following key terms were used: ‘electromagnetic field,’ ‘mobile phones,’ ‘mobile phone base stations,’ ‘watching TV,’ ‘using Internet,’ ‘miscarriage,’ ‘abortions,’ ‘spontaneous abortion,’ ‘early abortion’ and ‘late abortion’. All case–control and cohort studies that investigated the effect of EMF exposure on the risk of miscarriage were included without any restriction of language or time. Statistical analyses were done using Comprehensive Meta-Analysis software (version 2.0). A random-effects model was performed to calculate the overall effect size. A primary search revealed a total of 982 relevant studies; six articles (*N* = 3,187 participants) met the inclusion criteria for the meta-analysis. The results of the random-effects meta-analysis indicated that EMF exposure had a significant effect on miscarriage: rate ratio (RR) = 1.699; 95% confidence interval (CI): 1.121, 2.363 (*P* < 0.001); and heterogeneity (I^2^) = 84.55% (*P* < 0.001). The findings showed that pregnant woman who were exposed to high levels of EMF had an increased risk of miscarriage.

## Introduction

The term abortion refers to the termination of a pregnancy before the foetus has reached the stage of viability outside the uterus, which is usually around the 20th week of gestation (1). Spontaneous abortion is the most common complication of pregnancy (2); it occurs in about 30%–40% of all pregnancies (3). Abortion is a highly traumatic event that can have extensive psychological consequences for couples (4). In addition, bleeding and infection commonly occur after an abortion (5).

Spontaneous abortion occurs in about one-third of pregnancies (6). About half of all abortions are caused by abnormalities in the zygote, embryo, foetus or placenta; chromosomal abnormalities in the embryo account for almost 20% of all abortions. Chromosomal abnormalities that are caused by mutations, chromosomal translocation and chromosomal breaks can cause the abortion of euploid embryos, which are embryos that have a normal chromosomal composition. However, other causes of euploid abortions are not well known, although they may be related to maternal infectious diseases, high-risk pharmacological drugs, developmental disorders and environmental factors, such as smoking and alcohol consumption, the use of contraceptives, and exposure to environmental toxins, such as lead and arsenic (7, 8). During pregnancy, the health and living conditions of the mother can directly affect the foetus; in addition, the foetus is sensitive and vulnerable to various environmental factors (9, 10). Recognising the predisposing causes of abortion is an important women’s health issue that should be addressed to improve the quality of healthcare and the reproductive and sexual rights of women (11).

Electromagnetic waves are composed of electric and magnetic fields or electromagnetic fields (EMF), that are perpendicular to each other; they are generated from various devices that use electric and magnetic forces to operate (12). Given that electricity is an integral part of life, people are regularly exposed, both knowingly and unknowingly, to radiation from EMF produced by electrical devices, such as televisions, refrigerators, range hoods, microwaves, computers, mobile phones, power plants and transmission lines, powerhouses, medical diagnostic equipment, incubators and microscopes (13). Electromagnetic waves can affect living organisms (14). Berg (15) considered that the effect of EMF on the function of cells was determined by the intensity, duration of exposure and frequency of the electromagnetic waves.

Research centres, including the World Health Organization (WHO), have begun to pay close attention to the effects that exposure to EMF from generating devices and appliances have on human health (16). There are also concerns about the effects that EMF exposure have on pregnant women during the different stages of foetal growth. Several studies have been conducted on the effects of EMF exposure on pregnancy, although conflicting results have been reported regarding early foetal death, foetal abnormalities and behavioral changes in the infant after birth (12, 17).

Studies have been performed on pregnant animals. For example, one study on pregnant mice showed that exposure to very weak electromagnetic waves reduces the number of embryos that reach the morula stage, decreases the number of endothelial cells of the embryo and affects the trophectoderm (18). Exposure to EMF from a mobile telecommunication system was found to increase the risk of abortion in pregnant rats and to reduce the weight and body length of the foetus; exposure also caused disorders of the skeletal system, such as short or absent tails, lack of free ribs and lack of caudal vertebrae (19). Studies on pregnant women have shown that EMF emitted from microwaves, mobile phones and the internet can reduce amniotic fluid and increase the risk of abortion and death. Exposure to EMF can also have adverse effects on the growth and health of the foetus and can cause an increase in the volume, weight and atresia follicles in ovaries of children (12, 19–23).

A comprehensive study of the effects of exposure to EMF on pregnancy is necessary because of the increasing use of appliances and devices that generate EMF in homes, in industries and in the medical field; for example, mobile phones, which generate EMF, are used by billions of people around the world (24). The present systematic review and meta-analysis was performed to evaluate and summarise the results of various studies on the effect that exposure to electromagnetic waves has on abortion.

## Methods

### Inclusion and Exclusion Criteria

This review included all case-control and cohort studies that investigated the effects of EMF exposure on the risk of miscarriage. There were no restrictions in terms of language or time. Articles on all types of abortion, including spontaneous abortions, early abortions and late abortions, were included; however, studies on animals were excluded. Studies that assessed a women’s exposure to EMF during pregnancy as the first or secondary outcome were included in the meta-analysis. Our review was not limited to a specific description of EMF and, thus, exposure from using mobile phones and mobile phone base stations, TVs, and the Internet were included. Studies that investigated exposure to extremely low-frequency EMF and exposure through occupations were also included.

### Data Sources and Search Strategy

Our initial search involved using the Medline search strategy. A systematic search was then conducted on the PubMed, CINAHL, Scopus, Web of Science, Google Scholar and Cochrane Library databases for all relevant articles published up until August 2021. A search of all reference lists from recognised articles, meta-analyses and review articles and other relevant studies was performed using the following key terms: ‘electromagnetic field’, ‘mobile phones’, ‘mobile phone base stations’, ‘watching TV’, ‘using Internet’, ‘miscarriage’, ‘abortions’, ‘spontaneous abortion’, ‘early abortion’ and ‘late abortion’.

### Study Selection and Data Extraction

After a general search, all potentially suitable papers were collected by two researchers. All titles and abstracts of the papers were assessed and unsuitable papers were excluded. The remaining articles were then carefully evaluated by two authors (MI and MAM), independently. In the case of an article in which there was no agreement between the reviewers after a discussion, a third author would comment. The selected articles were arranged according to a predetermined checklist, which included the first author, year of publication, reference, country, study design, sample size, investigated population, age range, follow-up period, adjusted variables, main findings and quality score.

### Statistical Methods

All statistical analyses were performed using Comprehensive Meta-Analysis software version 2.2.064. A level of significance of *P* < 0.05 was used. To assess the statistical heterogeneity between the studies, Cochran’s Q test, with a significance level of *P* ≤ 0.1 and the heterogeneity (I^2^) test, with a significance level of ≥ 50%, were used. Publication bias was assessed by a visual inspection of Begg’s funnel plots and the use of asymmetry tests (i.e. Egger’s and Begg’s tests). In this study, a meta-analysis was done using the random-effects model. A sensitivity analysis was performed by removing studies one by one and checking the *P*-value of the pooled effect (i.e. the leave-one-out method in sensitivity analysis).

### Quality Assessment and Data Extraction

Quality assessment was performed using the Newcastle-Ottawa Scale (NOS). The NOS was developed to assess the quality of non-randomised studies in terms of their design, content and ease of use for the interpretation of the results of a meta-analysis.

## Results

**Figure 1:**
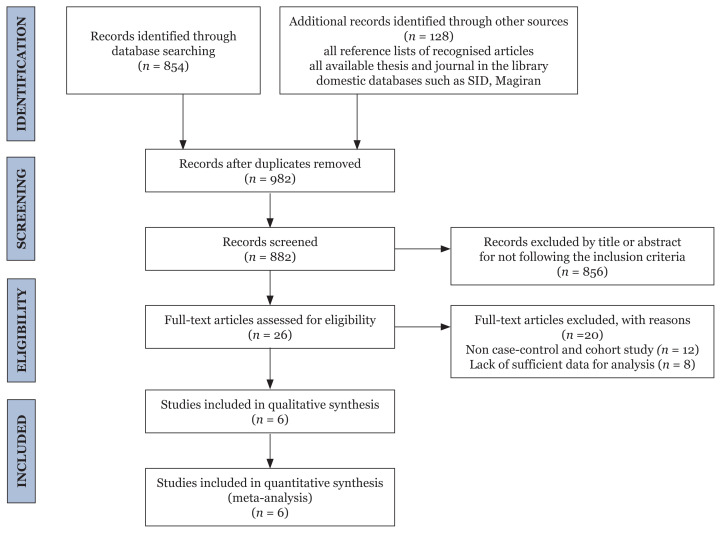
PRISMA chart of study selection process

In this study, an electronic search of databases identified 982 potentially relevant studies. After duplicated articles were removed, 882 articles were screened for inclusion. Two reviewers excluded 856 articles after reviewing their titles and abstracts. Finally, after the full text of 26 published studies on the effect of EMF on the risk of miscarriage were reviewed, six articles, which included 3,187 pregnant women, met the inclusion criteria of this study. The PRISMA flow diagram of the selected studies is shown in Figure 1. The main characteristics of the eligible studies are described in Table 1.

From the six studies that were reviewed, two followed the cohort design and four were case-control studies. Three of the studies were performed in Iran, while three were from the United States (25–30).

### Assessment of the Quality of Studies

To assess the quality of the studies, a star system was developed based on three broad perspectives: i) the selection of the study groups; ii) the comparability of the groups and iii) the identification of either the exposure or the outcome of interest for the case–control or cohort studies, respectively (Table 2). The minimum and maximum scores of the star system ranged between 0 and 9. Articles that scored ≥ 6 were considered to be high quality articles.

### Meta-Analysis Results

**Figure 2:**
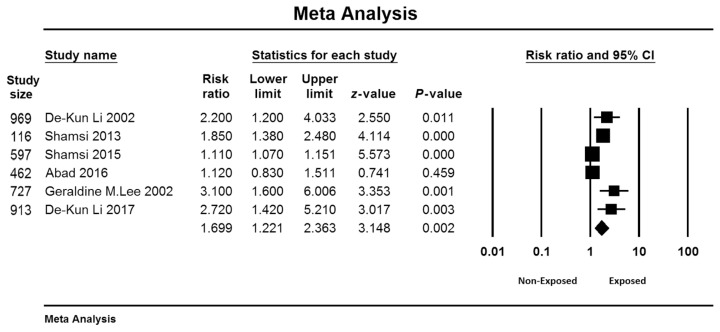
Effect of EMF on the risk of miscarriage

Figure 2 shows the forest plots that summarise the meta-analysis of the EMF effect on miscarriage. The results of the random-effects meta-analysis indicated that EMF had a significant effect on miscarriage: rate ratio (RR) = 1.699; 95% confidence interval (CI): 1.221, 2.363.

### Heterogeneity, Publication Bias and Sensitivity Analysis of Included Studies

**Figure 3:**
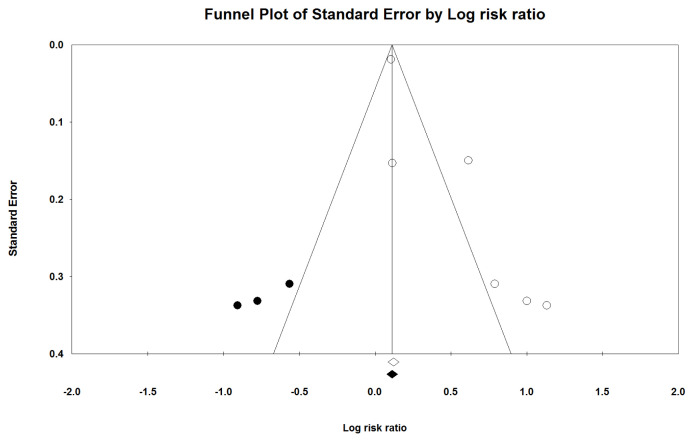
The funnel plot of the effect of EMF on the risk of miscarriage

The heterogeneity between the included studies was significant: Q-statistic *P* < 0.001 and I^2^ = 84.55%. The symmetrical funnel plot shown in Figure 3 demonstrates a significant bias in the publications based on Egger’s linear regression test (intercept = 2.515; standard error [SE] = 0.640.; 95% CI: 0.737, 4.294; *t* = 3.926; df = 4.00; two-tailed *P* = 0.01) and Begg’s rank correlation test (Kendall’s Tau with continuity correction = 0.40; z = 1.12; two-tailed *P*-value = 0.25). The trim-and-fill correction method by Duval and Tweedie resulted in the imputation of a potentially missing study and an adjusted effect size of 1.19 (95% CI: 0.87, 1.63). The fail-safe *N*-test indicated that 92.00 studies would be required to render the effect size as non-significant.

**Figure 4:**
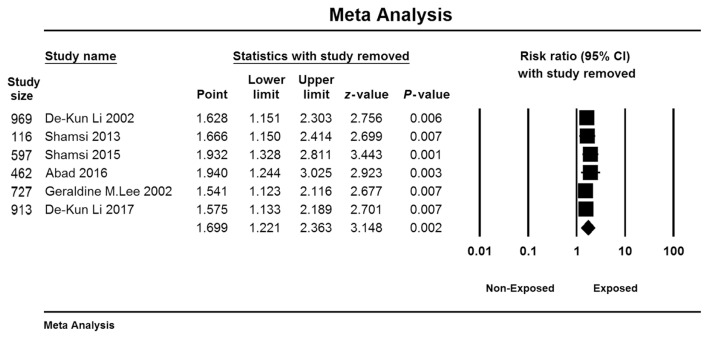
The results of the leave-one-out sensitivity analysis

The results of the sensitivity analysis showed that no study significantly influenced the results of the meta-analysis regarding the effect of EMF exposure on the risk of miscarriage (Figure 4).

## Discussion

This systematic review and meta-analysis study was performed to investigate the effects that exposure to EMF during pregnancy had on the risk of spontaneous abortion. Six articles were included in the meta-analysis; five studies confirmed the effect of exposure to electromagnetic waves on spontaneous abortion (25–30). The study by Abad et al. (27) indicated that, although women who were exposed to significant levels of electromagnetic waves had a high risk of miscarriage, the relationship was not confirmed by the Wald test. The lack of evidence may have been related to the small sample size of the study (27).

The present meta-analysis study showed that the risk of miscarriage in pregnant women who were exposed to EMF was 1.69 times higher than the risk for women who were not exposed. Ebadi et al. (31) showed that there was a significant relationship between exposure to low-frequency EMF (i.e. 3 Hz–3000 Hz) generated from sources in the home and the risk of miscarriage in pregnant women at < 14 weeks gestation. The researchers also found that the duration of mobile phone use during the day and the intervals between mobile phone use were associated with the risk of miscarriage (31).

Other studies have studied the effects that exposure to EMF have on pregnant animals. For example, Baharara et al. (32) exposed pregnant mice on the 14th day of gestation to waves from mobile phones for 6 h/day for 4 days; they also exposed 2-day-old mice. Their results showed that exposure to 940 MHz waves from mobile phones increased the number of micronuclei in the peripheral blood erythrocytes of the mothers and their babies; an increase in the number of micronuclei is a sign of chromosomal damage. These results confirmed the genotoxic effect of the waves (32).

Mobile phones emit radio frequency energy, which is a type of non-ionising radiation. The tissues in the human body that are closest to the antenna absorb this energy. This energy can interfere with magnetic fields in the human body and can cause disorders in the function of various organ systems in a developing foetus (20).

There are various theories about the possible link between EMF and abortion. For example, EMF may affect chemical interactions in cell membranes; they may decrease the permeability of cell membranes, which results in decreased cell connections; they may increase the number of free radicals in the body; they can impair mitotic divisions; and EMF may damage cell proteins and cause cell severance. Another theory is that the depth of penetration by EMF into tissues increases when fluids are low (33).

The risk of abortion from exposure to electromagnetic waves depends on the distance from the radiation source and the frequency of the waves (31). Another important factor that affects the relationship between exposure to electromagnetic waves and spontaneous abortion is the duration of exposure. In other words, can controlling the exposure to electromagnetic waves reduce the risk of abortion? Different studies have reported conflicting results in terms of the duration of exposure and the risk of abortion. For example, Li et al. (28) found strong evidence that exposure to a magnetic field over 16 mG may be associated with a risk of miscarriage. Their study showed that the RR associated with a magnetic field exposure of 16 mG was 2.2 (95% CI: 1.2, 4.0). The researchers also showed that the risk of miscarriage from exposure to magnetic waves was greater in early pregnancy (< 10 weeks) because the foetus was more sensitive to environmental factors (28).

In their case–control study, Lee et al. (29) found that exposure to high and frequent magnetic fields increased the risk of abortion in pregnant women enrolled in a medical care system in Northern California. The researchers stated that the risk of abortion increased with exposures above the 50th percentile level in the environment (29). In another study, researchers looked at mobile phone use and the risk of abortion in two groups of women: the case group of women had a spontaneous abortion at < 14 weeks and the control group of women were > 14 weeks gestation. They found that the average duration of mobile phone contact during the day, the location of the phone when not in use, the use of the phone for other applications, the specific absorption rate (SAR) and the mean effective SAR were significantly different between the two groups (26).

One of the limitations of the present study was the lack of access to relevant theses and unpublished studies, which could have contributed to the results of this study. One of the strengths of the present study was that the confounding variables that affected abortion were controlled in the results of all the studies in this meta-analysis. Therefore, it was possible to accurately determine the effect of electromagnetic waves on abortion.

## Conclusion

It is hoped that the results of this study will increase awareness of healthcare providers, such as gynaecologists and midwives, about the adverse effects of electromagnetic waves on pregnancy and the risk of miscarriage. In this way, pregnant women will be encouraged to protect themselves from exposure to electromagnetic waves. Pre-pregnancy and early pregnancy counseling on the proper way to limit exposure to electromagnetic waves from electrical appliances and devices, mobile phones and the internet may reduce the risk of spontaneous abortions.

## Figures and Tables

**Table 1:** Description and characteristics of studies included in the systematic review and meta-analysis

First author, year, reference	Country	Study design	Age range (years old)	Investigated variable	Investigated population	Follow-up period	Sample size		Main finding	Adjusted variables	Quality score

							Case/Exposure	Control/Non-exposure			
Shamsi-Mahmoudabadi, 2013 (25)	Iran	Case-control	18–35	Abortion	Pregnant women with gestational age 14 days or with an abortion < 14 weeks	2013	58	58	Extremely low frequency electromagnetic fields exposure is probably related to early spontaneous abortions.	Maternal and paternal ages, pre-pregnancy BMI, duration from last delivery, educational level, occupation and history of previous abortions or preterm labour.	9
Shamsi-Mahmoudabadi, 2015 (26)	Iran	Case-control	18–35	Abortion	Pregnant women with gestational age 14 days or with an abortion < 14 weeks	*N/A	292	308	use of mobile phones can be related to the early spontaneous abortions	Maternal and paternal ages, gravidity, pre-pregnancy BMI, family relationship	9
Abad (2016) (27)	Iran	Case-control	18–35	Spontaneous abortion	Pregnant women with gestational age < 14 weeks	*N/A	57	405	A significant likelihood of miscarriage in women who exposed to significant level of electromagnetic wave	Age, number of pregnancies, relatives of the spouse	8
Li 2002 (28)	California/USA	Cohort	*N/A	Early abortion	Pregnant women with gestational age at less than 10 weeks	1996–1998	159	810	There is strong evidence that exposure to a maximum magnetic field above a certain level (probably about ≥ 16 mG) during pregnancy is associated with a risk of miscarriage.	risk factors for miscarriage and other adverse pregnancy outcomes, occupational exposures to MF, daily activities during pregnancy	9
Lee (2002) (29)	California/USA	Case-control	At least 18 years old	Abortion	Pregnant women whit gestational age ≤ 14 weeks, spoke English or Spanish and were members of a California Kaiser Permanente Medical Care Programme	1990–1991	550	177	Gradient in miscarriage risk as the number of environments with exposures at or above the 50th percentile level increased.	Age, gestational age, history of abortion, race, smoking and alcohol and caffeine, BMI, income, type of home (multi-unit), nausea in the first trimester, education, physical health, the amount of physical activity	9
Li 2017 (30)	California/USA	Cohort	18 years old or older	Abortion	All pregnant women, aged 18 years old or older and residing in the participating bay area countries	*N/A	694	**219**	Women who were exposed to higher MF levels had 2.72 times the risk of miscarriage than those with lower MF exposure.	Maternal age, race, education, marital status, type of occupation in recent years, caffeine and smoking and alcohol use, parity, number of previous abortions, history of infertility, presence of vaginal bleeding and urinary tract infection since last menstrual period	9

Note:

* N/A = not available

**Table 2:** The NOS quality assessment of the included studies in this study

Cohort star template								

Study	Selection of cohorts				Comparability of cohorts	Outcome		

	Representativeness of the exposed cohort	Selection of the non-exposed cohort	Ascertainment of exposure	Demonstration that outcome of interest was not present at start of study	Comparability of cohorts on the basis of the design or analysis	Assessment of outcome	Was follow-up long enough for outcomes to occur	Adequacy of follow-up of cohorts
Li (28)	*	*	*	*	**	*	*	*
Li (30)	*	*	*	*	**	*	*	*

**Case-control star template**								

**Study**	**Selection of case and controls**				**Comparability of cases and controls**	**Exposure**		

	**Is the case definition adequate**	**Representativeness of the cases**	**Selection of controls**	**Definition of controls**	**Comparability of cases and controls on the basis of the design or analysis**	**Ascertainment of exposure**	**Same method of ascertainment for cases and controls**	**Non-response rate**

ShamsiMahmoudabadi (25)	*	*	*	*	**	*	*	*
ShamsiMahmoudabadi (26)	*	*	*	*	**	*	*	*
Lee (29)	*	*	*	*	**	*	*	*
Abad (27)	*	*	*	*	*	*	*	*

## References

[1] Cunningham FG, Leveno KJ, Bloom SL, Dashe JS, Hoffman BL, Casey BM (2018). Williams obstetrics.

[2] Mostakhdemin M, Shaiegan M, Kasraeian L, Khosravi A, Yari F, Shayegan S (2019). Antibody against human leukocyte antigens in female blood donors with and without previous abortion. Iran J Obstet Gynecol Infertil.

[3] Pereza N, Ostojić S, Kapović M, Peterlin B (2017). Systematic review and meta-analysis of genetic association studies in idiopathic recurrent spontaneous abortion. Fertil Steril.

[4] Matin M, Bashash D, Hasrak K, Baghestani A, Hamidpour M (2019). Frequency of protein C, S and antithrombin III deficiency and presence of antibodies of antiphospholipid syndrome in women with recurrent abortion. Iran J Obstet Gynecol Infertil.

[5] Haghdoost M, Mousavi S, Gol MK, Montazer M (2019). Frequency of *Chlamydia trachomatis* infection in spontaneous abortion of infertile women during first pregnancy referred to Tabriz University of Medical Sciences by nested PCR method in 2015. Int J Women Health Reprod Sci.

[6] Mitchell-Jones N, Gallos I, Farren J, Tobias A, Bottomley C, Bourne T (2017). Psychological morbidity associated with hyperemesis gravidarum: a systematic review and meta-analysis. Int J Obstet Gynae.

[7] Kim K, Sung HK, Lee K, Park SK (2019). Semiconductor work and the risk of spontaneous abortion: a systematic review and meta-analysis. Int J Environ Res Pub Health.

[8] Aradmehr M, Azhari S, Ahmadi S, Azmodeh E (2016). Relationship between delivery and neonatal factors with healing of episiotomy in primiparous women at Mashhad Omalbanin hospital in 2013. Iranian J Obstet Gynecol Infertil.

[9] Moosavinasab MS, Fahami F, Kazemi A (2018). The relationship between cognitive social theory and physical activity in pregnant women. Int J Pediatr.

[10] Ansari F, Akbari L, Kohan SH (2021). Evaluation of the effect of unfavorable working conditions on the incidence of spontaneous abortion in operating room staff and nurses. Armaghane Danesh.

[11] Nosrati F, Karimi F, Afiat M, Emami Moghadam Z (2021). An overview of the various dimensions of abortion in International Human Rights documents. Navidno.

[12] Hosseini E, Zia Z (2016). Effect of cell-phone radiation in pregnancy on serum levels of sexual hormones and dynastic cells in adult female offspring in rats. J Ardabil Univ Med Sci.

[13] Kaydani M, Panahi D, Saranjam B, Abdollahi M (2020). The effect of cellular phone microwave radiation on sperm fertility indices (count, motility, viability and morphology) in mice. J Health.

[14] Nazari M, Mofid M, Sadraee H, kaka G (2020). The effect of low frequency electromagnetic fields on anxiety behaviors and histomorphometry of inner pyramidal layer neurons of frontal cortex in adult male rat. Paramed Sci Military Health.

[15] Berg H (1999). Problems of weak electromagnetic field effects in cell biology. Bioelectrochem Bioenergy.

[16] Borhan F, Zavvar Reza J, Pandeh M, Fathi S (2017). Effects of exposure to Wi-Fi signals (2.45 GHz) on serum oxidative stress and single strand DNA breaks in peripheral blood lymphocytes of mice. J Shahid Sadoughi Univ Med Sci.

[17] Wdowiak A, Mazurek PA, Wdowiak A, Bojar I (2017). Effect of electromagnetic waves on human reproduction. Annals Agri Environ Med.

[18] Darabi MR, Bayat PD (2012). Effects of low electromagnetic field on mice embryos development. J Gorgan Uni Med Sci.

[19] El-Sayed A, Badr HS, Yahia R, Salem SM, Kandil AM (2011). Effects of thirty minute mobile phone irradiation on morphological and physiological parameters and gene expression in pregnant rats and their fetuses. *Afr J Biotechnology*.

[20] Jelodar G, Roudashtian M (2009). Effect of radiation leakage of microwave oven on pregnant mice. JBUMS.

[21] Hosseini Z, Moayyedi F, Dashti E (2019). Evaluation and prediction of the impact of parasite waves and cell phone use by pregnant mothers on the volume of amniotic fluid based on data mining algorithms. J Health Biomed Inform.

[22] Ren Y, Chen J, Miao M, Li D-K, Liang H, Wang Z (2019). Prenatal exposure to extremely low frequency magnetic field and its impact on fetal growth. Environ Health.

[23] Li D-K, Chen H, Ferber J, Hirst AK, Odouli R (2020). Association between maternal exposure to magnetic field nonionizing radiation during pregnancy and risk of attention-deficit/hyperactivity disorder in offspring in a longitudinal birth cohort. JAMA Netw Open.

[24] The World Bank Group [Internet] (2012). www.worldbank.org.

[25] ShamsiMahmoudabadi F, Ziaei S, Chahkandi T, Firoozabadi M, Kazemnejad A (2013). Exposure to extremely low frequency electromag-netic fields during pregnancy and the risk of spontaneous abortion: a case-control study. J Res Health Sci.

[26] ShamsiMahmoudabadi F, Ziaei S, Chahkandi T, Firoozabadi M, Kazemnejad A (2015). Use of mobile phone during pregnancy and the risk of spontaneous abortion. J Environ Health Sci Eng.

[27] Abad M, Malekafzali H, Simbar M, Mosaavi HS, Khoei EM (2016). Association between electromagnetic field exposure and abortion in pregnant women living in Tehran. Int J Reprod BioMed.

[28] Li D-K, Odouli R, Wi S, Janevic T, Golditch I, Bracken TD (2002). A population-based prospective cohort study of personal exposure to magnetic fields during pregnancy and the risk of miscarriage. Epidemiology.

[29] Lee G, Neutra R, Hristova L, Yost M, Hiatt R (2002). A nested case-control study of residential and personal magnetic field measures and miscarriages. Epidemiology.

[30] Li D-K, Chen H, Ferber J, Odouli R, Quesenberry C (2017). Exposure to magnetic field non-ionizing radiation and the risk of miscarriage: a prospective cohort study. Sci Rep.

[31] Ebadi A, Pournorouz H, Aghajanpour Mir SS, Raisian M, Ghobadi M, Habibian T (2020). Relationship between the exposure to magnetic fields during pregnancy and risk of abortion: a review article. Int J Pediatr.

[32] Baharara J, Hadad F, Shariatzade MA, Amirahmadi M (2011). The effects of cellular phone waves on thefrequency micronucleus in newborn and adult Balb/c mouse. Zahedan J Res Med Sci.

[33] Röösli M, Hug K (2011). Wireless communication fields and non-specific symptoms of ill health: a literature review. Wien Med Wochenschr.

